# Latin American consensus on psoriasis severity classification^☆^

**DOI:** 10.1016/j.abd.2024.09.010

**Published:** 2025-04-29

**Authors:** Angela María Londoño-García, Juan Raúl Castro-Ayarza, Manuel Darío Franco Franco, Cesar Fernando González Ardila, Gabriel Magariños, Enrique Salvador Rivas Zaldívar, Susan Martínez, Linda Ibatá, Julieth Carolina Castillo, Paola Jimena Cárdenas Rojas, Evelyn Giuliana Castro Vargas, Claudia Romina Contreras, Carolina Ivette Cortes Correa, Claudia de la Cruz Fernández, Andrés Chavarriaga Restrepo, Cristina Mariela Echeverria, André Vicente Esteves de Carvalho, Benjamín Hidalgo Matlock, Enrique Loayza, Matías Rafael Maskin, Ricardo Romiti, Fernando Valenzuela

**Affiliations:** a Dermatology Postgraduate Program, Faculty of Medicine, CES University, Medellin, Colombia; b Dermatology Postgraduate Program, Faculty of Medicine, National University of Colombia, Bogota, Colombia; c Dermatology Service, Medicarte IPS, Bogota, Colombia; d Private practice, Bogota, Colombia; e Dermatology Service, Dermatological Medical Center - Psoriahue, Buenos Aires, Argentina; f Research Department, Dermatological Medical Center DERMOS, Ciudad de Guatemala, Guatemala; g Epidemiology Department, EpiThink Health Consulting, Bogota, Colombia; h Dermatology Service, Medicarte IPS, Bogota, Colombia; i Dermatology Service, National Hospital Alberto Saboga, Callao, Peru; j Faculty of Medical Sciences, Hospital de Clínicas, National University of Asunción, Asuncion, Paraguay; k Dermatology Service, University Hospital of La Samaritana, Bogota, Colombia; l Dermatología, Clínica Dermacross, Santiago, Chile; m Rheumatology Service, CES Clinic, Medellin, Colombia; n Dermatology Service, Institute of Psychophysical Rehabilitation, Buenos Aires, Argentina; o Dermatology Service, Moinhos de Vento Hospital, Porto Alegre, Brazil; p Faculty of Medicine, University of Costa Rica, Latin University of Costa Rica, San Jose, Costa Rica; q Dermatology Service, Institute of Rheumatology, Hematology, and Dermatology, Guayaquil, Ecuador; r Dermatology Service, CEMIC, Dermatology Service, Buenos Aires Skin, Buenos Aires, Argentina; s Departamento de Dermatología, Universidad de São Paulo, São Paulo, Brasil; t Departamento de Dermatología, Universidad de Chile, Departamento de Dermatología, Universidad de los Andes, Chile

**Keywords:** Classification, Consensus, Delphi technique, Patient acuity, Psoriasis

## Abstract

**Background:**

There are different classifications of psoriasis based on its clinical presentation, impact on quality of life, requirements for specific treatments, and other patient- or physician-reported outcomes. However, the lack of unified definitions has led to the severity of the disease being underestimated. Standardizing the classification of psoriasis will promote a better approach to the disease and facilitate care by professionals.

**Objective:**

To present a consensus of experts in Latin America regarding the classification of psoriasis severity, based on the best available evidence and applicable to current medical practice in the region.

**Methods:**

An independent methodological team, together with a group of clinical dermatologists representatives from different Latin American countries, developed a consensus with a modified Delphi methodology based on a systematic review of the literature. This consensus includes the classification of psoriasis, tools to define the severity of psoriasis, and other considerations in evaluating patients with psoriasis.

**Results:**

Fifteen statements were formulated aimed at classifying the severity of cutaneous psoriasis and other forms of the disease, as well as tools to assess and define the severity of psoriasis and therapy considerations. Additionally, the consensus addresses implementation considerations.

**Conclusion:**

The results of this consensus constitute a solid basis for a standard classification terminology for the varied clinical forms of psoriasis and their therapeutic implications. The importance of maintaining a personalized therapeutic approach, adjusted to each country's available resources and administrative realities, is highlighted.

## Introduction

Psoriasis is a chronic multisystemic inflammatory disease that affects between 0.1% and 1.5% of the world’s population and is often associated with comorbidities such as psoriatic arthritis, metabolic syndrome, diabetes, cardiovascular disease, nephropathy, and bowel disease, among others.^1^ The classic cutaneous presentation of psoriasis consists of scaly, localized, or widespread erythematous plaques that affect patients' quality of life and are amenable to long-term treatment.^2^

Psoriasis can be classified according to its clinical presentation, impact on patients' lives, and the need for specific treatments. Some authors propose that psoriasis could be cutaneous or systemic.^1^ Other authors have described that psoriasis severity should include a combination of measures reported by the evaluator and the patient.^3^ In clinical practice, the severity of psoriasis is often classified into two or three categories according to different criteria. Several tools are often used to assess the severity of psoriasis, such as the Psoriasis Area and Severity Index (PASI), the affected Body Surface Area (BSA), the Physician's Global Assessment (PGA) and other instruments that measure the severity of the disease and the effectiveness of the treatments used.^4^ Other systems combine functional and psychosocial evaluations to obtain a more comprehensive assessment of the patient's condition and their degree of disease involvement.

Globally validated psoriasis severity categories are not currently recognized. Most reported classifications and definitions of disease severity and treatment response have been developed for clinical trials and have little use in clinical practice.^5^ The lack of uniform definitions regarding the classification of psoriasis is primarily due to the heterogeneity of disease presentation and the variability of assessment tools. In this scenario, the severity of the disease may be underestimated, so it is necessary to specify the evaluation of psoriasis considering aspects such as the appearance of lesions in specific locations, symptoms, extent of the disease, comorbidities, indicated treatment, and impact on quality of life.^6^

In Latin America, there are guidelines^7^ and consensuses^8^, ^9^, ^10^, ^11^ regarding the diagnosis and treatment of psoriasis, however, there is no uniform position on the classification of patients with psoriasis based on severity; this is especially relevant in unclear situations, such as when there is a discordance between clinical scales and the impact on quality of life or disability.^4^ Standardization of these concepts in psoriasis will promote better management of the disease and facilitate care by professionals.

Based on the best available evidence and medical expertise, this consensus offers definitions regarding the severity classification of psoriasis applicable to the current medical practice of dermatologists and rheumatologists in Latin America. This document considers the ethnic, social, cultural, and economic heterogeneity of the countries in the region, and its content should be adapted to the reality of each country and the individual circumstances of patients.

## Methods

### Consensus panel

An independent methodology team and a group of clinical dermatologists were part of the development group for this consensus. Representatives from Latin American countries (Argentina, Brazil, Chile, Colombia, Costa Rica, Ecuador, Guatemala, Paraguay, and Peru) completed the deliberative panel. Participants were selected based on their clinical experience in the management of psoriasis. The development group defined the consensus topics and guided the search and selection of studies and the validation of the evidence included in the analysis. The entire panel discussed the recommendations, voted, and defined the final consensus statements.

Prior to consensus development, participants agreed to participate actively and provided a declaration of interest.

### Evidence search

This consensus includes the classification of psoriasis, tools to define the severity of psoriasis, and other considerations when evaluating patients. A systematic literature review was conducted to identify the evidence to support the consensus analyses. The electronic databases MedLine and Embase were searched using strategies that included the following terms: “psoriasis” AND “disease severity” AND “assessment” OR “classification”. All searches were performed in October 2022 and updated in April 2023. Clinical Practice Guidelines (CPGs), consensus, and evidence-based recommendation documents were included. Depending on the need for information, other types of documents, such as narrative reviews, cross-sectional studies, and expert opinion articles, were also considered if they provided accurate information on the topic of interest. Abstracts and grey literature were considered if they contained information of interest. There were language restrictions (English and Spanish). There were no publication date restrictions. In addition, the authors searched the websites of scientific societies, compilers, and developers of clinical practice guidelines.

Once identified studies were collected, two reviewers independently assessed them for inclusion according to the pre-established selection criteria. References were screened for title and abstract, and later in full text as deemed necessary, with discrepancies between reviewers resolved by consensus. The documents selected for inclusion were graded with the Appraisal of Guidelines for Research and Evaluation (AGREE II) tool^12^ and the Checklist for Analytical Cross-Sectional Studies from the Joanna Briggs Institute^13^ according to the type of study. Supplementary Material 1 presents the search specifications, study selection, and quality of evidence.

### Delphi methodology

The modified Delphi methodology was the basis for formal consensus. The methodology group developed a questionnaire based on the statements extracted from the selected references. The development group reviewed and validated the content of the questionnaire. The final version included 52 items related to the following topics: factors influencing psoriasis severity, classification of psoriasis, assessment tools, and other considerations.

**Figure 1:**
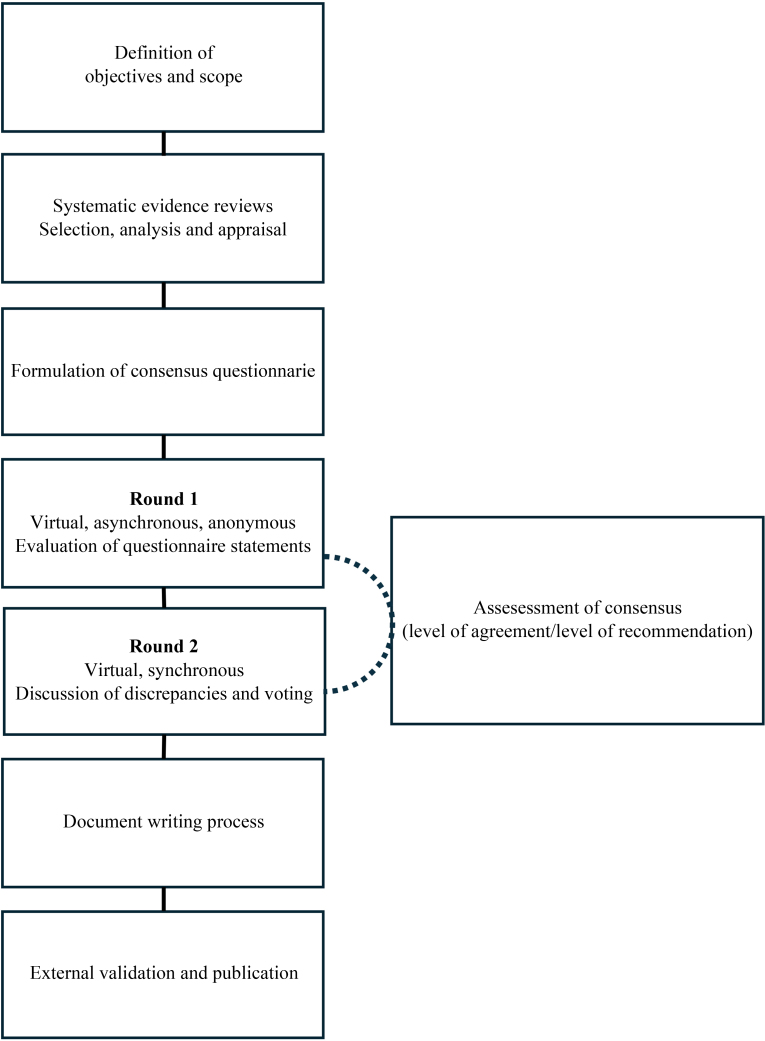
Consensus development on modified Delphi methodology.

The Delphi questionnaire was mailed to the entire panel of experts. Participants indicated their level of agreement with each statement using a 5-point Likert-type scale.^14^ The methodological team collected the results of the first round, which were analyzed to determine the level of agreement. Second, the panel discussed the items for which there was no clear consensus and the controversial aspects, followed by anonymous synchronous voting. Supplementary Material 2 presents the specifications of the Delphi process. Fig. 1 illustrates the consensus development process.

## Results and discussion

The evidence base for the consensus included seven clinical practice guidelines,^5^, ^7^, ^15^, ^16^, ^17^, ^18^, ^19^ eight consensuses,^8^, ^10^, ^11^, ^20^, ^21^, ^22^, ^23^, ^24^ three reviews,^25^, ^26^, ^27^ and two cross-sectional studies.^28^, ^29^ The evidence was analyzed and discussed as previously described, resulting in 15 guiding statements for psoriasis severity classification.

### Psoriasis severity classification

Table 1 describes the psoriasis severity classification.

**Table 1:** Psoriasis severity classification.

Consensus statements on definitions of psoriasis severity	Agreement level
**Cutaneous psoriasis**	
**Mild** cutaneous psoriasis is defined as:	85%
PASI < 5 with DLQI < 5, or	
Control with topical therapy	
It does not meet moderate or severe criteria.	
**Moderate** cutaneous psoriasis is defined as:	64%
PASI ≥ 5 and < 10 or	
DLQI 5 ≥ 5 and < 10, or	
Lack of response to topical treatment	
It does not meet severe criteria.	
**Severe** cutaneous psoriasis is defined as:	
PASI ≥ 10, or	84%
DLQI ≥ 10, or	85%
Erythrodermic or pustular variants, or	79%
Involvement of special localizations (e.g. face, palms, soles, genitals, scalp and nails), or	94%
Association with psoriatic arthritis, or	93%
Requirement of biological systemic therapy.	94%
**Scalp psoriasis**	
Scalp psoriasis is classified as severe when it affects more than 50% of the scalp and presents at least one of the following: severe erythema, severe scaling, extensive infiltration, moderate or severe itching, evidence of hair loss with scaling, or lesions extending beyond the scalp (e.g., forehead involvement).	82%
**Nail psoriasis**	
Few-nail disease is defined as affecting three or fewer nails.	71%

PASI, Psoriasis Area Severity Index; DLQI, Dermatology Life Quality Index.

#### Cutaneous psoriasis

Cutaneous psoriasis severity categories help clinicians make treatment decisions and are often used as inclusion criteria in clinical trials. The evidence about classification is heterogeneous; some authors propose three categories (mild, moderate, and severe),^10^, ^21^, ^25^, ^27^ while others propose only two (mild and moderate-severe).^8^, ^11^, ^15^ The inclusion of a “moderate” category is controversial. Although this category is not clearly defined, experts in practice recognize a group of patients who do not meet the criteria for maximum severity but do not respond to topical management or have greater clinical or quality-of-life impacts than those with mild disease.

Some experts believe clinical decisions do not change substantially between moderate and severe cases, while others see value in distinguishing these categories for better treatment access and description of cases. The classification in countries such as Argentina is typically divided into mild and moderate-severe categories. In contrast, experts in Brazil and Colombia believe that specifying a “moderate” category could facilitate the use of more specific treatments, given the breadth of the PASI scale. While a more precise categorization could reduce barriers to accessing treatment, it might also have therapeutic implications, such as imposing strict criteria for initiating treatments like methotrexate, conventional systemic therapy, or biosimilars. Additionally, differentiating between moderate and severe psoriasis could be useful for a more accurate description and analysis of cases by medical boards.

It is generally agreed that psoriasis severity classification should consider clinical evaluation (extent and inflammation) and quality of life, based on PASI and Dermatology Life Quality Index (DLQI) measurements, respectively. Involvement of special localizations (face, palms, soles, genitals, scalp, nails) and other forms (erythrodermic, and pustular variants) classify patients into the highest severity category. Comorbidities related to disease severity and treatment types (e.g., psoriatic arthritis, uveitis, inflammatory bowel disease) are also important considerations.

The classification of psoriasis is closely related to the type of treatment. Severe signs and symptoms, such as involvement of special localizations or intense pruritus, may require systemic treatment even if the PASI or BSA is < 10. A lack of response to topical treatment may lead to a moderate or severe psoriasis classification, depending on other factors of disease presentation and involvement (Table 2). Unless an individual assessment suggests otherwise, patients with moderate psoriasis should be prioritized for highly effective treatments, while for severe patients, this would be the only treatment option.

**Table 2:** Consensus statements on psoriasis therapy considerations.

Consensus statements on therapy considerations	Agreement level
Patients with psoriasis should be classified as topical or systemic therapy candidates.	82%
Candidates for systemic therapy are patients who meet at least one of the following criteria:	
BSA > 10%	
Involvement of special areas (face, palms, soles, genitals, scalp, or nails).	
Failure of topical therapy	88%
Evaluate associated comorbidities (psoriatic arthritis, uveitis, inflammatory bowel disease) or psoriatic arthritis to determine the treatment.	71%

BSA, Body Surface Area.

#### Other forms of psoriasis

According to an international consensus, nail psoriasis affecting three nails or fewer should be defined as a few-nail disease. This consensus also took into account the Nail Psoriasis Severity Index (NAPSI), defining mild nail disease as having a score of less than 20.^23^ However, the complexity of the NAPSI scale and the lack of evidence for severity classification limit its widespread use and, in the opinion of the panel, do not allow the establishment of thresholds for categorizing the severity of nail disease in clinical practice.

In scalp psoriasis, the PASI may underestimate severity because it is weighted by the percentage of body surface area affected. A modified version of the PASI, known as the Psoriasis Scalp Severity Index (PSSI), has been developed for more accurate assessment, although it is not widely used in clinical practice. The Physician's Global Assessment (PGA) is commonly used in medical practice for scalp psoriasis but lacks specific definitions for each severity level.^21^ Some experts have defined the severity of scalp psoriasis based on the extent of scalp involvement, as well as the presence and severity of erythema, scaling, itching, and the thickness of the lesions.^30^

The impact of scalp psoriasis on patients' well-being is increasingly recognized in evaluations. According to the panel of experts, the severity of scalp psoriasis should be assessed based on its effect on the patient. Generally, it is considered a severe condition when it affects more than half of the scalp and presents with one or more of the following: severe erythema, severe scaling, extensive infiltration, moderate to severe itching, and evidence of hair loss with flaking.

### Tools to assess and define psoriasis severity

Table 3 describes the tools used to assess and define the severity of psoriasis.

**Table 3:** Tools to assess and define psoriasis severity.

Consensus statements on psoriasis severity assessment	Agreement level
Assessment of the severity of psoriasis should include an objective assessment of the extent of the disease by the physician and a subjective assessment by the patient with regard to the impact on health-related quality of life.	94%
The PASI is the gold standard for assessing the clinical severity of plaque psoriasis because it is a widely validated and reproducible tool in adult patients with plaque psoriasis.	100%
The PASI should be assessed in patients with moderate to severe psoriasis as it correlates with other severity parameters such as the DLQI. Its percentage change helps assess the degree of improvement in psoriasis.	88%
Measurement of BSA can help assess the severity of psoriasis, stratify the patient's risk, and evaluate the response to treatment.	82%
The NAPSI is useful for assessing nail disease, functional or cosmetic impact, and treatment response.	71%
In specialized settings, and if practical in nonspecialized settings, it is recommended to use a validated tool to assess the impact of psoriasis on physical, psychological, and social well-being, such as the DLQI for adults or the CDLQI for children and young people.	100%
When using an assessment tool for a person with psoriasis, it is important to consider their age, disabilities, or limitations and provide support if necessary.	76%

PASI, Psoriasis Area Severity Index; CDLQI, Children's Dermatology Life Quality Index; DLQI, Dermatology Life Quality Index; NAPSI, Nail Psoriasis Severity Index; BSA, Body Surface Area.

The severity of the physical effects of psoriasis can be measured using various clinometric tools. Evidence supports the use of scales such as PASI, PGA, and BSA. Clinical practice guidelines^7^, ^16^, ^17^, ^19^ and consensus^20^ strongly recommend validated tools for assessing psoriasis severity.^18^ PASI measures the severity of skin lesions (erythema, scaling, and induration) and the involvement in four regions (head and neck, upper limbs, trunk, and lower limbs) with scores ranging from 0 to 72.^17^ It is commonly used to evaluate psoriasis severity and treatment response, with adequate correlation with other measures, but it has some disadvantages, such as complexity and low sensitivity to changes in less severe forms of psoriasis.^7^

The American Society of Dermatology recommends using the Body Surface Area (BSA) for mild psoriasis, while the PASI is recommended for moderate to severe psoriasis, either alone or in combination with PGA.^19^ The PGA has a close correlation with the PASI^16^ and is a validated tool for assessing physical severity with acceptable intraobserver and interobserver variability.^17^ However, the panel does not consider the use of PGA relevant for classifying the severity of psoriasis. On the other hand, approximately one-third of the clinical experts in this consensus routinely reported using BSA, especially in patients with psoriasis with small plaques.

In a cross-sectional study, ten dermatologists evaluated nine psoriasis patients twice with the PASI, PGA, and BSA scales to evaluate the correlation in the classification of psoriasis.^28^ Upon comparing the scales, it was found that the PGA had the highest interobserver reliability, while the BSA had the highest intraobserver reliability. The PASI showed intermediate values in terms of inter- and intraevaluation reliability. The authors concluded that none of the three assessment instruments showed an advantage over the others and recommended using several independent assessments simultaneously to evaluate the severity of psoriasis.

There are other tools that require further evaluation for assessing the severity of psoriasis, such as the Lattice System Physician's Global Assessment (LS-PGA), the Self-Administered Psoriasis Area Severity Index (SAPASI), and the Salford Psoriasis Index (SPI).^20^ Another questionnaire, called REFLETS (REFlective Evaluation of Psoriasis Efficacy of Treatment and Severity), was developed to evaluate the severity of psoriasis and the efficacy of treatment based on disease evolution, symptoms, lesion characteristics, and the impact of psoriasis. It classifies the disease as mild, moderate, or severe, with moderate to high correlations with the PASI (*r* = 0.35‒0.70) and the DLQI (*r* = 0.36‒0.82). However, as of the date of this manuscript, these results are only available in English and French.^29^

The heterogeneity of psoriasis makes it necessary to include the assessment of Health-Related Quality of Life (HRQL) and patient-reported outcome measures. The DLQI is the most frequently used, validated, and easy-to-apply tool in clinical practice.^17^ This tool is recommended by the NICE group^18^ to evaluate the impact of any type of psoriasis on physical, psychological, and social well-being. Other questionnaires used in research include the Short Form Health Survey (SF-36) and the Psoriasis Disability Index (PDI).^17^

In summary, the evaluation of patients with psoriasis should include measurement of both the clinical severity of the disease and its impact on the patient's quality of life. Both measures are important to ensure an appropriate approach to the disease.^17^ In addition to assessing symptoms such as itching, skin pain, burning, and bleeding from skin lesions, high-impact and difficult-to-treat sites (such as the face, scalp, palms, soles, folds, nails, and genitals) should also be evaluated.^20^ This comprehensive assessment should take into account any type of physical, visual or cognitive disability, language or communication difficulties, or other limitations, and should be adapted to the patient's age in order to obtain the most accurate results in estimating the severity of psoriasis.

### Considerations for implementation

Psoriasis is a heterogeneous disease that requires a comprehensive assessment of aspects such as body surface involvement, erythema, infiltration and desquamation of skin lesions, localization of lesions in sensitive areas (e.g., face, nails, genitalia, palmoplantar), impact on quality of life, response to topical or systemic treatments, and comorbidities. In 2009, the Latin American Society of Psoriasis^10^ established a holistic approach to assessing the severity of psoriasis, including other aspects, such as the patient's attitude towards the disease and the psychosocial impact, in addition to the usual ones. This consensus highlights the need to consider objective and subjective assessments of the burden of the disease from the perspective of the physician and the patient. Evaluations of psoriasis using tools that integrate these aspects are promising for improving the assessment of the severity of plaque psoriasis and the efficacy of treatment.

This document provides guidance on how to classify the severity of psoriasis according to current knowledge of the disease and suggests the preferred use of assessment tools that are available and widely used in the Latin American context. However, it is recognized that tools and assessment systems are constantly evolving to take into account all relevant aspects of the disease. Therefore, these recommendations should be updated in light of future knowledge of the disease, developments in practice, and the availability of resources for the assessment of psoriasis in the region.

### Conclusions

The heterogeneity in the presentation of psoriasis and the variations in the severity assessment have contributed to the lack of a unified position in its classification. The medical-scientific community in Latin America has recognized this need for standardization and has been motivated to approach the subject in order to facilitate communication among health professionals and to promote a more accurate approach to psoriasis in the region. This consensus document reflects the current understanding of the disease and its various clinical manifestations based on the best available evidence for the evaluation of patients with psoriasis. This panorama of recommendations highlights the importance of maintaining a personalized therapeutic approach adapted to the available resources and administrative realities of each country. Some aspects of psoriasis severity remain controversial, and the results of this consensus provide a solid basis for establishing a standard classification for the different clinical forms of psoriasis and their therapeutic implications, which is expected to impact disease management positively.

## External review and consensus update

A preliminary version of this manuscript, previously approved by the authors, was submitted for external peer review. The need to update this consensus should be evaluated in three years or sooner if necessary.

## Financial support

This consensus is endorsed by the Latin American Psoriasis Society (SOLAPSO) and the Colombian Group of Psoriasis and Immunodermatology (COLPSOR), affiliated with the Colombian Society of Dermatology (ASOCOLDERMA). It was developed thoroughly and independently, with transparency and impartiality. The funders did not participate in the development of the consensus, the decisions of the panel, or the final manuscript.

## Authors’ contributions

Angela María Londoño García: Conception and design; Analysis and interpretation of data; critical review of content, and final approval of the manuscript.

Juan Raúl Castro-Ayarza: Conception and design; Analysis and interpretation of data; critical review of content, and final approval of the manuscript.

Manuel Darío Franco: Conception and design; Analysis and interpretation of data; critical review of content, and final approval of the manuscript.

Cesar Fernando González Ardila: Conception and design; analysis and interpretation of data; critical review of content, and final approval of the manuscript.

Gabriel Magariños: Conception and design; Analysis and interpretation of data; critical review of content, and final approval of the manuscript.

Enrique Salvador Rivas Zaldívar: Conception and design; analysis and interpretation of data; critical review of content, and final approval of the manuscript.

Susan Martínez: Contributed to the critical review of the literature; writing the initial version of the manuscript; editorial review of the final manuscript; conception and design, analysis and interpretation of data; critical review of content, and final approval of the manuscript.

Linda Ibatá: Contributed to the critical review of the literature; writing the initial version of the manuscript; editorial review of the final manuscript; conception and design; analysis and interpretation of data; critical review of content, and final approval of the manuscript.

Julieth Carolina Castillo: Contributed to the critical review of the literature; writing the initial version of the manuscript; editorial review of the final manuscript, conception and design; analysis and interpretation of data; critical review of content, and final approval of the manuscript.

Paola Jimena Cárdenas Rojas: Conception and design; analysis and interpretation of data; critical review of content, and final approval of the manuscript.

Evelyn Giuliana Castro Vargas: Conception and design; analysis and interpretation of data; critical review of content, and final approval of the manuscript.

Claudia Romina Contreras: Conception and design; Analysis and interpretation of data; critical review of content, and final approval of the manuscript.

Carolina Ivette Cortes Correa: Conception and design; Analysis and interpretation of data; critical review of content, and final approval of the manuscript.

Claudia de la Cruz Fernández: Conception and design; analysis and interpretation of data; critical review of content, and final approval of the manuscript.

Andrés Chavarriaga Restrepo: Conception and design; analysis and interpretation of data, critical review of content, and final approval of the manuscript.

Cristina Mariela Echeverria: Conception and design; analysis and interpretation of data; critical review of content, and final approval of the manuscript.

André Vicente Esteves de Carvalho: Conception and design; analysis and interpretation of data; critical review of content, and final approval of the manuscript.

Benjamín Hidalgo-Matlock: Conception and design; analysis and interpretation of data; critical review of content, and final approval of the manuscript.

Enrique Fabian Loaiza Sánchez: Conception and design; analysis and interpretation of data; critical review of content, and final approval of the manuscript.

Matías Rafael Maskin: Conception and design; analysis and interpretation of data; critical review of content, and final approval of the manuscript.

Ricardo Romiti: Conception and design; analysis and interpretation of data; critical review of content, and final approval of the manuscript.

Fernando Valenzuela: Conception and design; analysis and interpretation of data; critical review of content, and final approval of the manuscript.

## Conflicts of interest

Angela Maria Londoño Garcia has been a speaker for Abbvie, Boehringer Ingelheim, Bristol, Eli Lilly, Janssen, Novartis, Pfizer.

Juan Raúl Castro Ayarza has been a speaker for AbbVie, Amgen, Eli Lilly, Janssen, Novartis, and Pfizer.

Manuel Darío Franco Franco has been a speaker for AbbVie, Amgen, Eli Lilly, Janssen, Novartis, Pharmalab, and Sanofi.

Cesar Fernando González Ardila has been a speaker for: AbbVie, Boehringer Ingelheim, Eli Lilly, Janssen, and Novartis.

Enrique Salvador Rivas Zaldivar has been a speaker for AbbVie and Novartis.

Paola Jimena Cardenas Rojas has been a speaker for AbbVie, Amgen, Elli Lilly, and Janssen.

Evelyn Giuliana Castro Vargas has been a speaker for AbbVie, Janssen and Tecnofarma.

Andrés Chavarriaga Restrepo has been a speaker for Amgen, Eli Lilly, Janssen, Novartis, Pfizer, Pharmalab.

Carolina Ivette Cortes Correa has been a speaker for Eli Lilly, Novartis, Bristol, Janssen.

Claudia de la Cruz Fernández has been a speaker or researcher for AbbVie, Boehringer Ingelheim, Bristol Myers Squibb, Eli Lilly, Janssen, Novartis, Pfizer, Sandoz, UCB Pharma.

Cristina Mariela Echeverria has been a speaker for AbbVie, Boehringer Ingelheim, Bristol Myers Squibb, Eli Lilly, Janssen, L'Oreal, Novartis, Pfizer, Sandoz, UCB Pharma.

André Vicente Esteves de Carvalho has been a speaker for AbbVie, Boehringer Ingelheim, Eli Lilly, Janssen and Novartis.

Benjamin Hidalgo Matlock has been a researcher for Cutera and Novartis.

Enrique Fabian Loaiza Sánchez has been a speaker for Janssen, Medicament, and Novartis.

Ricardo Romiti has been a speaker for AbbVie, Boehringer, Eli Lilly, Janssen, LEO Pharma, Novartis, Teva and UCB.

Fernando Valenzuela has been a speaker for AbbVie, Boehringer Ingelheim, Eli Lilly, Jannsen, LEO, and Novartis.

Other authors do not declare conflicts of interest.
