# Supplementary material for: Latin American consensus on psoriasis severity classification
Source: An Bras Dermatol. 2025 Apr 29;100(3):539–47. doi: 10.1016/j.abd.2024.09.010 (PMC12234208; doi:10.1016/j.abd.2024.09.010)
Supplement: Supplementary file 1 [file mmc1.docx]

**ABD-D-24-00513**

**Supplementary Material**

**Supplementary Material 1** Search, selection and quality of evidence.

| A) Evidence search strategies | |
| --- | --- |
| **Database, Platform: Medline, Pubmed** | |
| **Date of search: October 2022** | |
| **Search strategy (results)** | |
| 1. psoriasis [Title], "Humans, English, Spanish, Adult: 19+ years, Young Adult: 19‒24 years, Adult: 19‒44 years, Middle Aged + Aged: 45+ years, Middle Aged: 45‒64 years, Aged: 65+ years, 80 and over: 80+ years", (""psoriasis"" [Title]) AND ((humans [Filter]) AND (english [Filter] OR spanish [Filter]) AND (alladult [Filter] OR youngadult [Filter] OR adult [Filter] OR middleaged [Filter] OR middleaged [Filter] OR aged [Filter] OR 80andover [Filter])) "11,527" | |
| 2. (((((disease severity assessment [Title/Abstract]) OR (severity psoriasis [Title/Abstract])) OR (psoriasis assessment [Title/Abstract])) OR (psoriasis assessment [Title/Abstract])) OR (psoriasis classification [Title/Abstract]), "Humans, English, Spanish, Adult: 19+ years, Young Adult: 19‒24 years, Adult: 19‒44 years, Middle Aged + Aged: 45+ years, Middle Aged: 45‒64 years, Aged: 65+ years, 80 and over: 80+ years", "(""disease severity assessment"" [Title/Abstract] OR ""severity psoriasis"" [Title/Abstract] OR" "assessment evaluation" "[Title/Abstract] OR" "psoriasis assessment" "[Title/Abstract] OR" "psoriasis classification" "[Title/Abstract]) AND (humans [Filter]) AND (english [Filter] OR english [Filter]) AND (alladult [Filter] OR youngadult [Filter] OR adult [Filter] OR middleaged [Filter] OR middleaged [Filter] OR aged [Filter] | |
| 3. ((((((Severity of Illness Index [MeSH Terms]) OR (Severity of Illness Index [Text Word])) OR (Index [Title/Abstract])) OR (Measure [Title/Abstract])) OR (Title/Abstract])) OR (quality of life [MeSH Terms])) OR (clinimetric [Title/Abstract]), "Humans, English, Spanish, Adult: 19+ years, Young Adult: 19-24 years, Adult: 19-44 years, Middle Aged + Aged: 45+ years, Middle Aged: 45‒64 years, Aged: 65+ years, 80 and over: 80+ years", "(""severity of illness index "[MeSH Terms] OR" "severity of illness index" "[Text Word] OR" "Index" "[Title/Abstract] OR" "measure" "[Title/Abstract] OR" "score" "[Title/Abstract] OR" "quality of life"" [MeSH Terms] OR ""clinimetric"" [Title/Abstract]) AND ((humans [Filter]) AND (english [Filter] OR spanish [Filter]) AND (alladult) | |
| 4. (## 1) AND (#2)) AND (#3), "Humans, English, Spanish, Adult: 19+ years, Young Adult: 19‒24 years, Adult: 19‒44 years, Middle Aged + Aged: 45+ years, Middle Aged: 45‒64 years, Aged: 65+ years, 80 and over: 80+ years", "(""psoriasis"" [Title] AND (""humans"" [MeSH Terms ] AND (""english"" [Language] OR ""spanish"" [Language]) AND (""adult"" [MeSH Terms] OR ""young adult"" [MeSH Terms] OR ""adult"" [ MeSH Terms: noexp] OR (""middle aged"" [MeSH Terms] OR ""aged"" [MeSH Terms]) OR ""middle aged"" [MeSH Terms] OR ""aged"" [MeSH Terms] OR ""aged, 80 and over"" [MeSH Terms])) AND ((""disease severity assessment"" [Title/Abstract] OR ""severity psoriasis"" [Title/Abstract] OR ""assessment evaluation"" [Title/Abstract] OR". | |
|  | |
| **Database, Platform: Ovid** | |
| **Date of search: October 2022** | |
| **Search strategy (results)** | |
| 1) psoriasis.ti. | 30105 |
| 2) exp psoriasis/ | 46351 |
| 3) 1 or 2 | 49755 |
| 4) Disease severity assessment.tw. | 110 |
| 5) (disease adj2 severity adj2 assessment).tw. | 782 |
| 6) (PSORIASIS adj severity).ab. | 921 |
| 7) (severity adj2 psoriasis).ab. | 2173 |
| 8) (evaluation adj2 psoriasis).ab. | 49 |
| 9) (assessment adj2 psoriasis).ab. | 445 |
| 10) (classification adj2 psoriasis).ab. | 29 |
| 11) Severity of Illness Index.sh. | 268915 |
| 12) (index adj3 psoriasis).tw. | 1220 |
| 13) (measure adj3 psoriasis).tw. | 78 |
| 14) (score adj3 psoriasis).tw. | 213 |
| 15) Quality of life.sh. | 252187 |
| 16) Clinimetric.ab. | 1028 |
| 17) 4 or 5 or 6 or 7 or 8 or 9 or 10 | 3351 |
| 18) 11 or 12 or 13 or 14 or 15 or 16 | 504520 |
| 19) 3 and 17 and 18 | 1679 |

| **B) Flowchart of the search, screening and selection of evidence (PRISMA) from databases** |
| --- |
| **** |

| **C) Quality assessment of selected studies.** | | |
| --- | --- | --- |
| **Cross-sectional studies.** Checklist for Analytical Cross-Sectional Studies, JBI. | | |
| **First author / Year** | **Bożek 2017** | **Gilet H 2015** |
| 1. The selection criteria were clearly defined | Yes | Yes |
| 2. Participants and locations were described in detail | Yes | Yes |
| 3. Exposure was measured with valid and reliable instruments | Yes | Yes |
| 4. The measurement criteria were standardized | Yes | Yes |
| 5. Confounding variables were identified | No | No |
| 6. Were strategies established to address confounding factors | No | No |
| 7. Outcomes were measured with valid and reliable instruments | Yes | Yes |
| 8. Statistical analysis was appropriate | Yes | Yes |

| **Clinical practice guidelines** Appraisal of Guidelines for Research and Evaluation (AGREE II). | | | | | | | |
| --- | --- | --- | --- | --- | --- | --- | --- |
| **Domain** | **CPG Sign [1]** | **CPG Spain [2]** | **CPG Malaysia [3]** | **CPG Italy [4]** | **CPGNICE [5]** | **CPG Colombian [6]** | **CPG AAD [7]** |
| Domain 1 |  |  |  |  |  |  |  |
| Scope and Objective | 77.8% | 61.1% | 88.9% | 44.4% | 100.0% | 100.0% | 83.3% |
| Domain 2 |  |  |  |  |  |  |  |
| Participation of those involved | 83.3% | 50.0% | 94.4% | 44.4% | 100.0% | 100.0% | 83.3% |
| Domain 3 |  |  |  |  |  |  |  |
| Rigor in the elaboration | 93.8% | 58.3% | 89.6% | 52.1% | 100.0% | 100.0% | 64.6% |
| Domain 4 |  |  |  |  |  |  |  |
| Clarity of presentation | 100.0% | 88.9% | 100.0% | 55.6% | 100.0% | 100.0% | 94.4% |
| Domain 5 |  |  |  |  |  |  |  |
| Applicability | 95.8% | 12.5% | 87.5% | 25.0% | 100.0% | 100.0% | 33.3% |
| Domain 6 |  |  |  |  |  |  |  |
| Editorial independence | 100.0% | 100.0% | 100.0% | 100.0% | 100.0% | 100.0% | 100.0% |

CPG, Clinical Practice Guideline.

**References**

1. Burden AD, Hilton Boon M, Leman J, Wilson H, Richmond R, Ormerod AD; Guideline Development Group. Diagnosis and management of psoriasis and psoriatic arthritis in adults: summary of SIGN guidance. BMJ. 2010;341:c5623.

2. Puig L, Carrascosa JM, Carretero G, de la Cueva P, Lafuente-Urrez RF, Belinchón I, et al.; Spanish Psoriasis Group of the Spanish Academy of Dermatology and Venereology. Spanish Evidence-Based Guidelines on the Treatment of Psoriasis With Biologic Agents, 2013. Part 1: On Efficacy and Choice of Treatment. Actas Dermosifiliogr. 2013;104:694-709.

3. Choon S, Chan L, Choon SE, Jamil A, Chin CL, Cheng CH, et al. Malaysian Clinical Practice Guideline for the Management of Psoriasis Vulgaris: Summary of recommendations for management in primary healthcare setting. Malays Fam Physician. 2014;9:16-21.

4. Gisondi P, Altomare G, Ayala F, Bardazzi F, Bianchi L, Chiricozzi A, et al. Italian guidelines on the systemic treatments of moderate-to-severe plaque psoriasis. J Eur Acad Dermatol Venereol. 2017;31:774-90.

5. NICE. Psoriasis: Assessment and Management. nice.org.uk/cg153. (2017).

6. Asociación Colombiana de Dermatología y Cirugia Dermatológica. Guías Basadas En La Evidencia Para El Manejo de La Psoriasis En Colombia. https://revistasocolderma.org/content/guias-basadas-en-la-evidencia-para-el-manejo-de-la-psoriasis-en-colombia (2018).

7. Elmets CA, Korman NJ, Prater EF, Wong EB, Rupani RN, Kivelevitch D, et al. Joint AAD-NPF Guidelines of care for the management and treatment of psoriasis with topical therapy and alternative medicine modalities for psoriasis severity measures. J Am Acad Dermatol. 2021;84:432-70.

**Supplementary Material 2** Delphi consensus process.

| For the consensus rounds, the questionnaire constructed from the evidence was shared with the experts. The statements were assessed using a Likert-type scale, as indicated by the “appropriate use method” developed by the RAND Corporation and the University of California at Los Angeles (UCLA).[1] The response categories are located in three areas (1‒2 disagreement; 3 neither agreement nor disagreement; 4‒5 agreement). Each panelist scored each statement according to the degree of agreement with the statement. |
| --- |
| The first round of responses to the questionnaire was carried out “blind” to the judgment of the other members of the panel through a virtual questionnaire. The information from this round was analyzed using frequency distributions and measures of central tendency, classifying each item in consensus scenarios according to the level of agreement and the direction of the recommendation, as shown in the following table: |
| **Analysis Parameters for the Consensus Voting Method (Delphi)** |
| **Agreement level** |
| **Consensus:** If the extreme points of the range of responses are located within any of the three regions of the scale zone (1‒2; 3; 4‒5). |
| If the extreme points of the range are located within two consecutive regions or <30% of panelists scored outside the region that contains the median. |
| **No consensus**: The scores of a minimum of 30% of panelists are in the region of 1‒2, and a minimum of 30% in the region of 4‒5 or the interquartile range ≥3. |
| **Indeterminate**: Statements in which there is neither agreement nor disagreement. |
| **Direction of recommendation** |
| **Agreement:** Median in interval 4‒5 and without disagreement. |
| **Disagreement**: Median in interval 1‒2 and without disagreement. |
| **Doubtful**: Median in 3 or no consensus. |
| A second round was carried out synchronously and anonymously in a debate session, during which new evaluations were carried out (round 2) and recommendations were reformulated. All experts participated in the two decision rounds. |

1. Holey EA, Feeley JL, Dixon J, Whittaker VJ. An exploration of the use of simple statistics to measure consensus and stability in Delphi studies. BMC Med Res Methodol. 2007;7:52.
